# Diagnostic Sensitivity of Saliva and Other Respiratory Tract Samples of SARS-CoV-2 Variants in Patients with COVID-19

**DOI:** 10.1128/spectrum.03076-22

**Published:** 2023-03-28

**Authors:** Merlin Jayalal Lawrence Panchali, Choon-Mee Kim, Yu-Mi Lee, Jun-Won Seo, Da Young Kim, Na Ra Yun, Dong-Min Kim

**Affiliations:** a Department of Internal Medicine, College of Medicine, Chosun University, Gwangju, Republic of Korea; b Premedical Science, College of Medicine, Chosun University, Gwangju, Republic of Korea; Quest Diagnostics

**Keywords:** SARS-CoV-2, COVID-19, delta, omicron, saliva

## Abstract

Severe acute respiratory syndrome coronavirus 2 (SARS-CoV-2) variants continue to emerge during the ongoing coronavirus disease 2019 (COVID-19) pandemic. Contrasting studies on the omicron variant have demonstrated higher viral loads in different clinical specimens, which is consistent with its high transmissibility. We investigated the viral load in clinical specimens that were infected with the SARS-CoV-2 wild-type, delta, and omicron variants, and we analyzed the diagnostic accuracy of upper and lower respiratory specimens for these variants. We performed nested reverse transcription (RT)-polymerase chain reaction (PCR), targeting the spike gene and sequencing for variant classification. RT-PCR was performed using upper and lower respiratory specimens, including saliva from 78 COVID-19 patients (wild-type, delta, and omicron variants). A comparison of the sensitivity and specificity, using the area under the receiver operating characteristic curve (AUC) values from the *N* gene, showed that the omicron variant saliva samples had a higher sensitivity (AUC = 1.000) than did the delta (AUC = 0.875) and the wild-type (AUC = 0.878) variant samples. The sensitivity of the omicron saliva samples was greater than that of the wild-type nasopharynx and sputum samples (*P *< 0.001). The viral loads of the saliva samples containing the wild-type, delta, and omicron variants were 8.18 × 10^5^, 2.77 × 10^6^, and 5.69 × 10^5^, respectively, which did not differ significantly (*P = *0.610). Statistically significant differences were not observed in the saliva viral loads between vaccinated and nonvaccinated patients who were infected with the omicron variant (*P = *0.120). In conclusion, omicron saliva samples had higher sensitivity than did wild-type and delta samples, and the viral load did not significantly differ between vaccinated and nonvaccinated patients. Further research is necessary to elucidate the mechanisms underlying the sensitivity differences.

**IMPORTANCE** Owing to the vast heterogeneity of the studies focused on the correlation between the SARS-CoV-2 omicron variant and COVID-19, accurate comparisons of the specificity and sensitivity of samples and associated outcomes are still inconclusive. Moreover, limited information is available on the leading causes of infection and the factors that are associated with the conditions that underlie the spread of infection. Although several studies have contributed important knowledge regarding infectious specimens, the impact of saliva samples remains unknown. This study showed that the sensitivity of the omicron variant saliva samples was higher than that of the wild-type nasopharyngeal and sputum samples. Moreover, neither vaccinated nor nonvaccinated patients who were infected with the omicron variant showed any significant differences in SARS-CoV-2 viral loads. Hence, this study is an important step toward understanding how saliva sample results are correlated with other specimen results, regardless of the vaccination status of patients who are infected with the SARS-CoV-2 omicron variant.

## INTRODUCTION

Coronavirus disease 2019 (COVID-19) is caused by severe acute respiratory syndrome coronavirus 2 (SARS-CoV-2) (1). Since the beginning of the COVID-19 pandemic, multiple SARS-CoV-2 variants have evolved; representative specific variants of concern include the Pango lineages B.1.1.7 (alpha), B.1.351 (beta), P.1 (gamma), B.1.617.2 (delta), and B.1.1.529 (omicron). Alpha was the first variant found in Kent, United Kingdom, and it was 30 to 40% more transmissible than was the wild-type SARS-CoV-2 (2). The beta variant was first identified in the Republic of South Africa during the fall of 2020, with 21 mutations, 9 of which were present in the spike protein gene (3). The delta variant was first identified in India, in October of 2020, and it has increased transmissibility with higher viral loads and a higher risk of hospitalization and mortality (4). Another variant, namely, omicron, was first reported in the Republic of South Africa in November of 2021, with numerous mutations conferring increased transmissibility (5). The omicron variant has 30 substitutions in the spike protein, relative to those observed in the previous variants (6).

Multiple upper and lower respiratory specimens, including throat swabs, oropharyngeal swabs, saliva, nasopharyngeal swabs, sputum, and bronchial fluid, have been used to diagnose COVID-19. Lower respiratory tract specimens are known to have higher viral loads, and nasopharyngeal swabs remain the gold standard specimen for SARS-CoV-2 diagnosis, but negligible false-negative and variable positive rates have been reported (7–10). Although previous studies have monitored the viral shedding and usefulness of saliva samples (11), these sensitivity studies of saliva samples were performed before the emergence of the delta and omicron variants. A nucleic acid amplification test with saliva was reported to be similar to nasopharyngeal swabs, and it was suggested that saliva can be used as an alternative specimen for SARS-CoV-2 detection (12). However, the sensitivity and specificity of saliva samples for different variants are not fully understood.

In the current study, we measured viral loads from clinical specimens obtained from patients with COVID-19 (infected with the SARS-CoV-2 wild-type, delta, and omicron variants). A reverse transcription-quantitative polymerase chain reaction (RT-qPCR)-based assay, targeting the *N*, *E*, and *RdRp* genes, was used to quantify the viral loads. In a previous study, saliva and midturbinate swab samples were preferred for omicron variant detection, suggesting changes in omicron’s tissue tropism (13). We measured the viral loads in upper (oropharyngeal, nasopharyngeal, and saliva) and lower (sputum) respiratory tract specimens as well as the diagnostic specificity and sensitivity. We also measured the differences in viral loads between vaccinated and nonvaccinated patients who were infected with the delta and omicron variants.

## RESULTS

### Viral load measurements differ from variants and sample sources.

**FIG 1:**
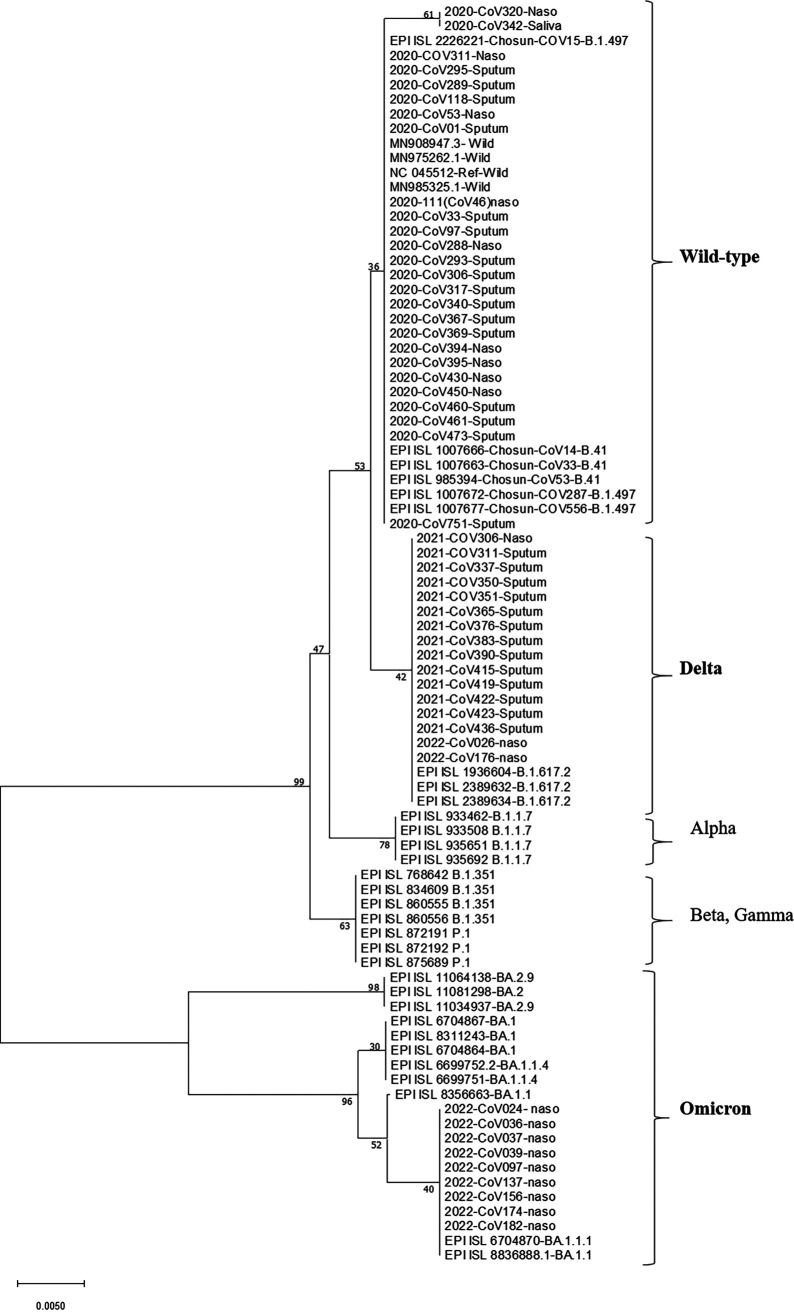
SARS-CoV-2 phylogeny. The phylogenetic tree was constructed by using the spike gene nested RT-PCR sequences. Variants clustered with the expected reference sequences from the GISAID database.

A total of 312 specimens, including nasopharynx, oropharynx, sputum, and saliva (78 each) specimens, were collected from 78 patients with COVID-19 (wild-type, delta, and omicron variants) within the first 3 days of hospital admission. The specimens were analyzed for the presence of SARS-CoV-2 RNA via RT-qPCR. Nested RT-PCR, targeting the spike gene, was performed for variant classification, and the positive PCR products were sequenced and phylogenetically analyzed. In addition, samples that were collected from patients with COVID-19 before the emergence of variants of concern, such as alpha, beta, and gamma, as well as variants that were closely related to the Wuhan sequences, were considered to be wild-type variants so as to differentiate them from the delta and omicron variants. When first-wave samples were submitted for NGS between May of 2020 and May of 2021, most South Korean samples were identified as B.1.497, which was present only in Korea. All of the positive amplicon sequences clustered well with the reference wild-type, delta, and omicron sequences (Fig. 1).

All of the samples were analyzed for the *N* gene; however, to reduce costs, only the nasopharyngeal and sputum specimens were analyzed also for the *E* and *RdRp* genes. The Ct threshold cutoff value was set to 40 for all samples. A higher viral load was observed in the nasopharyngeal and sputum samples of the wild-type, delta, and omicron variants using the *NP* gene target. Similar viral loads were observed in the nasopharyngeal and sputum samples using the *E* and *RdRp* genes. Consistent with our previously published results (14), the saliva samples exhibited better sensitivity than did the oropharynx samples. We analyzed the viral loads of nasopharyngeal, oropharyngeal, sputum, and saliva samples for the wild-type, delta, and omicron variants, using a nonparametric approach to an analysis of variance (ANOVA) to identify differences in the distribution of the viral load between the variants and between the sample sources. The distributions of *E* and *RdRp* in the sputum samples were significantly different for the delta and omicron variants. Similarly, the mean distributions were assessed for the different variants, and this was followed by Scheffe’s *post hoc* analysis. The distributions of the viral load for the delta and omicron samples were significant by the ANOVA but were not significant by the *post hoc* analysis. We conclude that the omicron viral loads, which were measured using the *NP* gene, were not significantly different from the wild-type and delta loads (Table 1).

**TABLE 1:** Viral loads, sensitivity, and specificity as a function of the viral variant and sample source^*a*^

Viral strain	Target gene	Wild			Delta			Omicron			*P* value for viral load		
		*NP*	*E*	*RdRp*	*NP*	*E*	*RdRp*	*NP*	*E*	*RdRp*	*NP*	*E*	*RdRp*
Nasopharynx	Viral load	3.75 × 10^6^	8.26 × 10^8^	8.47 × 10^8^	2.27 × 10^7^	1.17 × 10^9^	1.63 × 10^9^	1.20 × 10^7^	1.58 × 10^8^	1.51 × 10^8^	0.537	0.310	0.369
	Sensitivity/Specificity (AUC)	81.08/100 (0.905)	75.68/100 (0.878)	72.97/100 (0.865)	100/100 (1.000)	95.24/100 (0.976)	95.24/100 (0.976)	100/100 (1.000)	100/100 (1.000)	100/100 (1.000)			
Sputum	Viral load	1.03 × 10^7^	3.42 × 10^8^	5.56 × 10^8^	2.07 × 10^7^	2.47 × 10^8^	4.88 × 10^8^	1.91 × 10^7^	1.59 × 10^8^	1.38 × 10^8^	0.282	0.049	0.010
	Sensitivity/Specificity (AUC)	94.59/100 (0.973)	64.86/100 (0.824)	62.16/100 (0.865)	95.24/100 (0.976)	90.48/100 (0.952)	90.48/100 (0.952)	100/100 (1.000)	95.00/100 (0.975)	95.00/100 (0.975)			
Oropharynx	Viral load	6.69 × 10^4^	NA	NA	3.89 × 10^6^	NA	NA	4.48 × 10^4^	NA	NA	0.058	NA	NA
	Sensitivity/Specificity (AUC)	70.27/100 (0.838)	NA	NA	100/100 (1.000)	NA	NA	80/100 (0.900)	NA	NA			
Saliva	Viral load	8.18 × 10^5^	NA	NA	2.77 × 10^6^	NA	NA	5.69 × 10^5^	NA	NA	0.610	NA	NA
	Sensitivity/Specificity (AUC)	75.68/100 (0.878)	NA	NA	75.0/100 (0.875)	NA	NA	100/100 (1.000)	NA	NA			
*P* value for viral load	ANOVA	0.288			0.033			0.020					
	Scheffe’s	0.43^ac^. 0.45^ad^			0.11^ac^. 0.10^ad^			0.061^ac^. 0.127^ad^					

a Samples were collected between the first three days of hospital admission. A cycle threshold (cutoff) of 40 (C_t_ = 40) was used for the *NP* gene target. For *E* and *RdRp*, either a Kogene Kit or an SD Kit was utilized, according to the manufacturer’s instructions. AUC, area under the curve; NA, not applicable.

The sensitivity and specificity of the wild-type, delta, and omicron variant samples were assayed using receiver operator characteristic curves. The sensitivity and specificity of the N gene, using Ct 40 for the wild-type sputum samples, were 94.6% and 100%, respectively, with an area under the curve (AUC) of 0.973. For the nasopharyngeal samples, the sensitivity and specificity were 81.1% and 100% (AUC = 0.905), respectively. For the *E* gene, the sensitivity and specificity were 64.9% and 100% (AUC = 0.824) as well as 75.7% and 100% (AUC = 0.878) for the sputum and nasopharyngeal specimens, respectively. For *RdRp*, the sensitivity and specificity were 62.2% and 100% (AUC = 0.865) as well as 73% and 100% (AUC = 0.865) for the sputum and nasopharyngeal samples, respectively. Similarly, the sensitivity and specificity for the saliva samples using *N* were 75.7% and 100% (AUC = 0.878) for the wild-type, 75.0% and 100% (AUC = 0.875) for the delta variant, and 100% and 100% (AUC = 1.000) for the omicron variant, respectively (Table 1).

**FIG 2:**
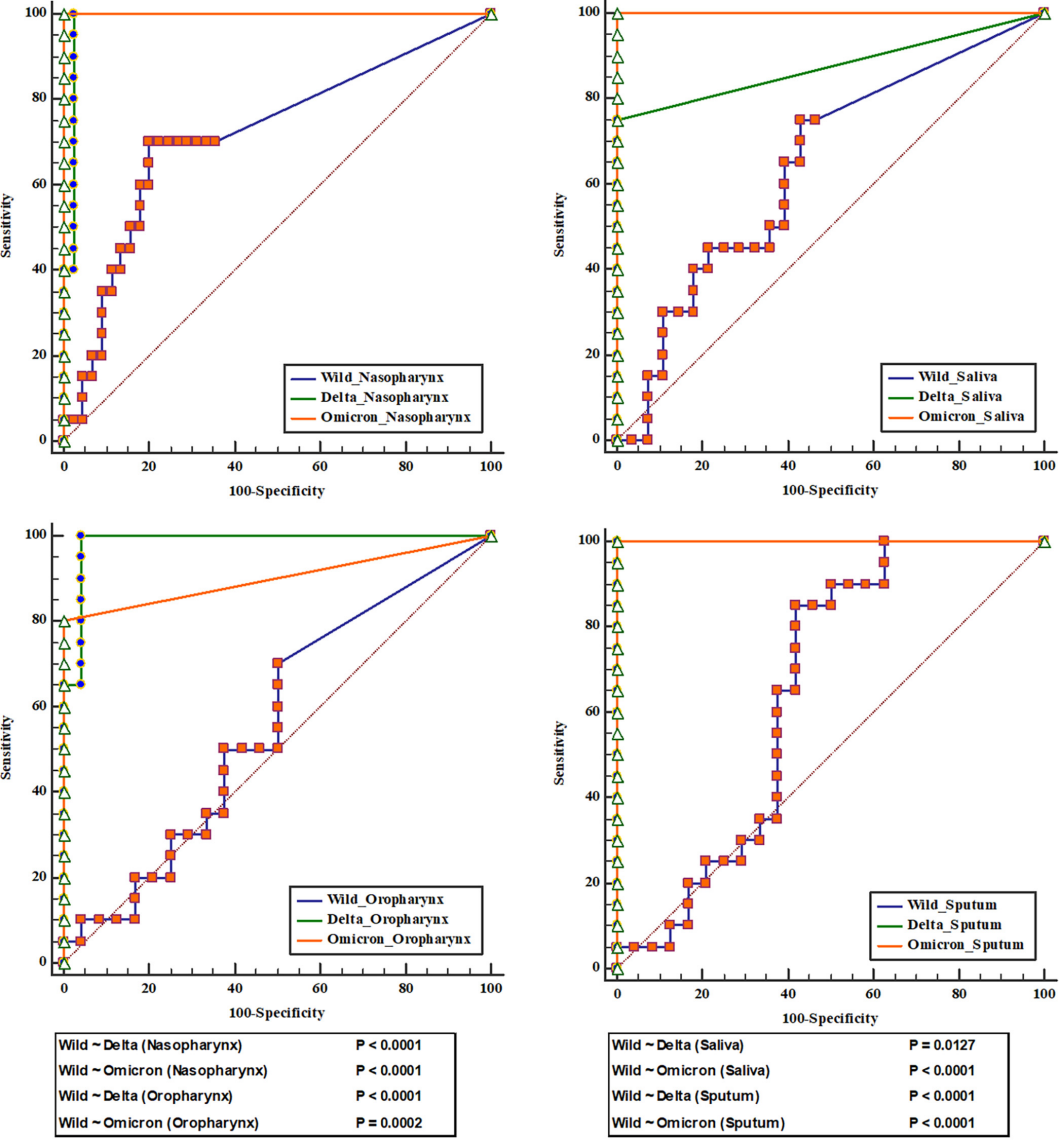
Specificity and sensitivity of the RT-qPCR for the SARS-CoV-2 wild-type and variants. Comparisons of the specificity and sensitivity are shown for each sample at Ct 40.

We assessed accuracy by using the *N* gene and receiver operator characteristic curves. The comparisons of the wild-type (AUC = 0.718) versus delta (AUC = 0.987) nasopharynx (*P < *0.001), wild-type (AUC = 0.718) versus omicron (AUC = 1.000) nasopharynx (*P < *0.001), wild-type (AUC = 0.558) versus omicron (AUC = 0.900) oropharynx (*P < *0.001), wild-type (AUC = 0.642) versus delta (AUC = 0.875) saliva (*P < *0.0127), omicron (AUC = 1.000) versus delta saliva (*P = *0.0118), wild-type (AUC = 0.652) versus delta (AUC = 1.000) sputum (*P < *0.001), and wild-type versus omicron (AUC = 1.000) sputum (*P < *0.001) were clinically significant (Fig. 2).

To evaluate the sensitivity of the saliva samples, we compared the nasopharyngeal and sputum samples to the saliva samples for all variants. The nasopharyngeal and sputum samples of the wild-type and delta variants exhibited higher sensitivity than did the saliva samples. We also observed that the omicron saliva samples had a higher sensitivity than did the wild-type saliva, nasopharynx, and sputum samples (*P < *0.001) (Fig. S1). For further confirmation, we compared the wild-type versus delta, wild-type versus omicron, and delta versus omicron samples (Fig. S2). The omicron saliva samples had a higher sensitivity than did the wild-type or delta variant saliva samples (*P < *0.001).

The mean viral loads of the wild-type, delta, and omicron variant saliva samples were 8.18 × 10^5^, 2.77 × 10^6^, and 5.69 × 10^5^, respectively, which were not significantly different (*P* = 0.61) (Table 1). Next, we measured viral copy number variation in vaccinated and nonvaccinated patients. None of the patients who were infected with the wild-type virus had been vaccinated, since the vaccine was not yet available in South Korea. The viral loads of the saliva samples, using the *N* gene, in vaccinated and nonvaccinated patients with the delta variant were 1.57 × 10^6^ and 3.03 × 10^6^ (*P = *0.43), respectively (Table 2). For omicron, the viral loads, using the *N* gene, in the saliva, sputum, and oropharyngeal samples of vaccinated (6.33 × 10^5^, 2.33 × 10^7^, and 5.72 × 10^4^, respectively) and nonvaccinated (4.49 × 10^5^, 1.31 × 10^7^, and 2.97 × 10^4^, respectively) patients were not significantly different. Although most patients with omicron had been vaccinated, there was no significant difference in viral load, according to vaccination status. Hence, further study is necessary to confirm the effects of vaccination.

**TABLE 2:** Viral RNA copy numbers, according to vaccinated and nonvaccinated patients^*a*^

Variant	Target gene	Nasopharyngeal viral load		*P* value	Sputum viral load		*P* value	Oropharyngeal viral load		*P* value	Saliva viral load		*P* value
		Vaccinated (N)	Nonvaccinated (N)		Vaccinated (N)	Nonvaccinated (N)		Vaccinated (N)	Nonvaccinated (N)		Vaccinated (N)	Nonvaccinated (N)	
Wild	*NP*	NA	3.75 × 10^6^ (30)		NA	1.03 × 10^7^ (35)		NA	6.69 × 10^4^ (27)		NA	8.18 × 10^5^ (28)	
	*E*	NA	8.26 × 10^8^ (30)		NA	3.42 × 10^8^ (35)		NA	NA		NA	NA	
	*RdRp*	NA	8.47 × 10^8^ (30)		NA	5.56 × 10^8^ (35)		NA	NA		NA	NA	
Delta	*NP*	2.96 × 10^7^ (4)	2.24 × 10^7^ (17)	0.030	1.68 × 10^7^ (4)	2.15 × 10^7^ (17)	0.031	4.97 × 10^6^ (4)	3.68 × 10^6^ (17)	0.148	1.57 × 10^6^ (4)	3.03 × 10^6^ (17)	0.092
	*E*	7.85 × 10^7^ (4)	2.19 × 10^9^ (17)	0.051	8.91 × 10^8^ (4)	1.78 × 10^8^ (17)	0.012	NA	NA		NA	NA	
	*RdRp*	1.44 × 10^8^ (4)	2.89 × 10^9^ (17)	0.032	2.46 × 10^9^ (4)	3.40 × 10^8^ (17)	0.04	NA	NA		NA	NA	
Omicron	*NP*	1.07 × 10^7^ (13)	1.48 × 10^7^ (7)	0.016	2.33 × 10^7^ (13)	1.31 × 10^7^ (7)	0.045	5.72 × 10^4^ (13)	2.97 × 10^4^ (7)	0.008	6.33 × 10^5^ (13)	4.49 × 10^5^ (7)	0.088
	*E*	2.45 × 10^8^ (13)	7.01 × 10^7^ (7)	0.094	2.22 × 10^8^ (13)	7.42 × 10^7^ (7)	0.031	NA	NA		NA	NA	
	*RdRp*	8.73 × 10^7^ (13)	4.85 × 10^8^ (7)	0.078	7.19 × 10^7^ (13)	4.59 × 10^8^ (7)	0.048	NA	NA		NA	NA	

a *P*-values for the comparisons between vaccinated and nonvaccinated patients, based on *t* tests.

## DISCUSSION

Marais et al. reported that the omicron variant had increased viral shedding in the saliva than in the nasopharynx, indicating a higher diagnostic performance of saliva samples; they suggested that omicron had changed its tropism to favor upper respiratory tract tissue (13). In a previous study, we analyzed the sensitivity and specificity of multiple clinical specimens in which the sputum was superior to the nasopharyngeal, saliva, and oropharyngeal specimens, but all of the virus samples were of the wild type (14). In another study, we assayed viral kinetics, comparing them with symptoms, treatment, and disease severity; we found that the viral load was significantly higher in patients who had received steroid therapy (15). In the present study, we measured differences in the viral loads of vaccinated and nonvaccinated patients. We also measured the viral loads of variant virus strains and different sample sources. A study performed in the United Kingdom showed that higher omicron viral loads may increase transmission via aerosolization (16). However, we observed no significant differences in viral loads between the omicron, delta, and wild-type SARS-CoV-2 variants. Our results are consistent with those of another previous study, which show that a higher viral load did not support the more rapid spread of the omicron variant, with the omicron viral load being lower than that of delta, thereby suggesting that the rapid spread of omicron might be a consequence of it bypassing the immunity generated by previous infection or vaccination (17). The infectiousness of omicron may not be linked with the higher viral loads that were measured in the upper or lower respiratory tract specimens because omicron has a lower viral RNA peak and a shorter clearance phase than does the delta variant; moreover, the authors suggested that the decreased viral load may be a result of vaccination (18). Another recent study showed that omicron infection is not triggered or enhanced by transmembrane serine protease 2 (TMPRSS2); instead, the enhancement is primarily mediated by endocytic pathways. Hence, these differences in infective pathways may have significant clinical implications for the omicron variant (19). A recent computational docking study showed that the omicron spike protein RBD residues 468 to 473 were more disordered, which possibly improves its binding to ACE2 to increase infectivity (20). In another study, the spike-ACE2 complex structure revealed new salt bridges and hydrogen bonds with changes to residues R493, S496, and R498. Neutralization assays showed that increased antibody evasion, together with stronger interactions with ACE2, is likely contributing to the more rapid spread of the omicron variant (21).

With respect to vaccination, the correlation of the viral load in vaccinated individuals with that in patients who were infected with the delta variant was lower than that in unvaccinated patients, who had a 4.5-fold increase in viral load, suggesting that omicron does not have an increased viral load, which implies that another mechanism may contribute to the speed of omicron infection (22). We also observed no significant difference in viral loads between vaccinated and nonvaccinated patients. A recent report suggested that for omicron, saliva samples are diagnostically superior to nasopharyngeal and oropharyngeal samples (23). Similar results were reported from a community study of wide-scale screening for COVID-19 (24).

Our study has several limitations, including a small number of vaccinated and nonvaccinated subjects and the fact that it was a single-center study. Therefore, further research is necessary to elucidate the mechanism underlying diagnostic sensitivity for omicron variants. In addition, as we observed no significant differences in the omicron viral loads between vaccinated and nonvaccinated patients, further studies with larger sample sizes will provide better statistical power.

In conclusion, our study demonstrates that saliva samples of the omicron variant have a higher sensitivity than do the delta and wild-type saliva samples, even though there is no significant difference in the viral copy numbers among the wild-type, delta, and omicron variants, irrespective of the vaccination statuses of the patients. Our comparisons of variants showed that for omicron, saliva samples provided better sensitivity than did wild-type or delta variant nasopharyngeal and sputum specimens.

## MATERIALS AND METHODS

### Ethics statement.

The study was approved by the Institutional Review Board (IRB) of Chosun University Hospital (IRB 2020-02-011-003), South Korea. Written informed consent was obtained from all of the participants.

### Participants.

We enrolled 78 patients who were infected with the wild-type, delta, or omicron SARS-CoV-2 variants from February of 2020 to December of 2021 at Chosun University Hospital. All of the participants were clinically confirmed to be infected by using several diagnostic methods, including (i) cell culture, (ii) a more than 4-fold increase in SARS-CoV-2 antibody titer or seroconversion using indirect immunofluorescence and enzyme-linked immunosorbent assays (25–27), or (iii) sequencing using an in-house nested RT-PCR targeting the spike gene or next-generation sequencing (NGS). In addition, RT-qPCR targeting the *N*, *E*, and *RdRp* genes was performed for all of the nasopharyngeal and sputum samples, whereas only *N* was targeted for the other samples. A total of 45 nasopharyngeal, 24 sputum, 24 oropharyngeal, and 28 saliva samples collected from healthy subjects with no history of COVID-19, no detection of SARS-CoV-2 antigens, and no clinical symptoms were used as negative-control samples. Written informed consent was obtained from the participants, and this study was approved by the Institutional Review Board (IRB) of Chosun University Hospital, South Korea (IRB 2020-02-011-003).

### Sampling and RNA extraction.

Nasopharyngeal and oropharyngeal swabs were collected directly by a physician using commercial UTM kits containing 1 mL of viral transport medium (Noble Bio, Seoul, South Korea). 200 μL aliquots were employed for RNA extraction. Sputum and saliva samples were self-collected by patients in collection tubes and were diluted with 1 mL of phosphate-buffered saline. Then, they were mixed via vortexing and centrifuged (200 × *g*, 1 min). A 200 μL aliquot of supernatant was subjected to RNA extraction. Viral RNA was extracted using a Real-prep Viral DNA/RNA Kit (Biosewoom, Seoul, South Korea) with full automation (Real-Prep system, Biosewoom).

### Reverse transcription-quantitative PCR (RT-qPCR).

RT-qPCRs targeted the *N* (nucleocapsid protein), *E* (envelope protein), and *RdRp* (RNA-dependent RNA polymerase) genes. For the *N* gene PCR, the primers and probes were designed in-house: nCov-N_572F (5′-GCAACAGTTCAAGAAATTC-3′), nCov-N_687R (5′-CTGGTTCAATCTGTCAAG-3′), and nCov-N_661P (5′-FAM-AAGCAAGAGCAGCATCACCG-BHQ1-3′). For the *E* and *RdRp* gene amplification, Kogene Kits (Kogene Biotech Co., Ltd., Seoul, South Korea) or SD Kits (SD Biotechnologies Co., Ltd., Seoul, South Korea) were used for diagnostic purposes, according to the manufacturer’s instructions (14). The cycle threshold (Ct) values were set to ≤40 for the reference gene and were set as the cutoff for positivity, as previously described (14). The viral loads were quantified using standard curves for all three genes. The Ct values were converted to log_10_ RNA copies/mL.

### Nested reverse transcription-PCR (nested RT-PCR) and variant detection.

For variant identification, nested RT-PCR was performed, targeting the spike gene, using complementary DNA (cDNA) and the following primer sets the for first and second rounds: first, nCoV_S-635F 5′-TAGTGCGTGATCTCCCTC-3′ and nCoV_S-2200R 5′-TCTTGGTCATAGACACTGG-3′; second, nCoV_S-860F 5′-CTGTAGACTGTGCACTTGAC-3′ and nCoV_S-1980R 5′-GAGTTGTTGACATGTTCAGC-3′. cDNA was synthesized by using SuperScript VILO MasterMix (Invitrogen, Carlsbad, CA, USA) and viral RNA, according to the manufacturer’s protocol. A commercial mutation identification kit (PowerChek SARS-CoV-2 S-gene Mutation Detection Kit) from Kogene Biotech was used for further identification. Positive amplicons were directly sequenced and analyzed using BLAST (NCBI, National Institutes of Health, Rockville, MD, USA), with the construction of a phylogenetic tree for variant confirmation. The partial sequence from the nested RT-PCR was 1.2 kb in length. After sequence alignment, all of the sample amplicons were cut to 960 bp. ClustalX (version 2.0; www.clustal.org/) and the Tree Explorer program 10 (DNASTAR, Madison, WI, USA) were used to construct a phylogenetic tree using these sequences.

NGS was performed commercially for only a few samples, and the sequences were deposited in GISAID. The Molecular Evolutionary Genetics Analysis (MEGA 11) software package was used to construct a phylogenetic tree using the NGS sequences.

### Statistical methods.

Quantitative data are expressed as means ± standard deviations or as medians (ranges) and percentages. The chi-square or Fisher’s exact tests were used for comparing categorical variables, when appropriate. The Mann-Whitney nonparametric test was utilized for comparing continuous variables, when appropriate. The sensitivity and specificity of predictive values and accuracy are presented as percentages. *P* values of <0.05 were considered to be indicative of a statistically significant result. Sensitivity and specificity were measured as areas under the receiver operator characteristic curves (AUC). For nonparametric tests to assess the viral load distribution, an analysis of variance (ANOVA) that was followed by a *post hoc* test was utilized. All of the statistical analyses were performed using the MedCalc software package (Ostend, Belgium) (28).

### Data availability.

The data will be available to the editors on request for a short period.
