# Supplementary material for: Diagnostic Sensitivity of Saliva and Other Respiratory Tract Samples of SARS-CoV-2 Variants in Patients with COVID-19
Source: Microbiol Spectr. 2023 Mar 28;11(2):e03076-22. doi: 10.1128/spectrum.03076-22 (PMC10100734; doi:10.1128/spectrum.03076-22)
Supplement: Supplemental file 1 — Supplemental material. Download spectrum.03076-22-s0001.pdf, PDF file, 0.7 MB [file spectrum.03076-22-s0001.pdf]

Supplementary Materials

Table of contents

Investigators…………………………………………………………..………………………………………………2
Supplementary Figures……………………………………………………………………..…………………………………4

Site of Principal Investigators

College of Medicine, Chosun University, Gwangju,
Republic of Korea

Investigators

Merlin Jayalal Lawrence Panchali,[1†] Choon-Mee Kim,[2†] Yu-Mi Lee,[1] Jun-Won Seo,[1] Da Young Kim,[1] Na Ra Yun,[1] and Dong-Min Kim[1]*

[1] *Department of Internal Medicine, College of Medicine, Chosun University, Gwangju, Republic of Korea*

[2] *Premedical Science, College of Medicine, Chosun University, Gwangju, Republic of Korea*

Supplementary Figures

Figure S1. Variation in sensitivity for viral strain and sample source.

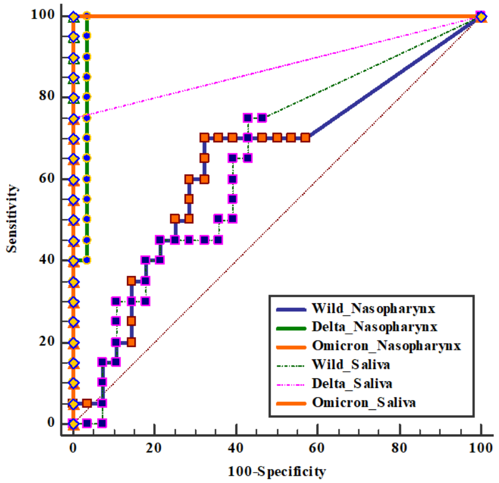

Wild nasopharynx ~ Delta nasopharynx	P < 0.0001
Wild nasopharynx ~ Omicron nasopharynx	P < 0.0001
Wild nasopharynx ~ Delta saliva	P = 0.0028
Wild nasopharynx ~ Omicron saliva	P < 0.0001
Delta nasopharynx ~ Wild saliva	P < 0.0001
Omicron nasopharynx ~ Wild saliva	P < 0.0001
Omicron nasopharynx ~ Delta saliva	P = 0.0118
Wild saliva ~ Delta saliva	P = 0.0127
Wild saliva ~ Omicron saliva	P < 0.0001
Delta saliva ~ Omicron saliva	P = 0.0118

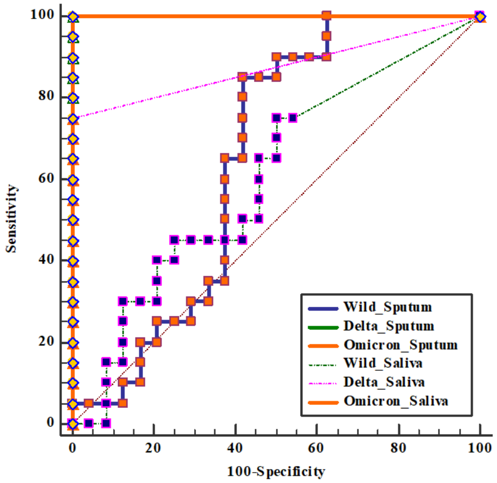

Wild sputum ~ Delta sputum	P < 0.0001
Wild sputum ~ Omicron sputum	P < 0.0001
Wild sputum ~ Delta saliva	P = 0.0232
Wild sputum ~ Omicron saliva	P < 0.0001
Wild sputum ~ Omicron saliva	P < 0.0001
Delta sputum ~ Wild saliva	P < 0.0001
Delta sputum ~ Delta saliva	P = 0.0118
Omicron sputum ~ Wild saliva	P < 0.0001
Omicron sputum ~ Delta saliva	P = 0.0118
Wild saliva~ Delta saliva	P = 0.0062
Wild saliva~ Omicron saliva	P < 0.0001
Delta saliva~ Omicron saliva	P = 0.0118

Figure S2. Pairwise sensitivity comparisons.

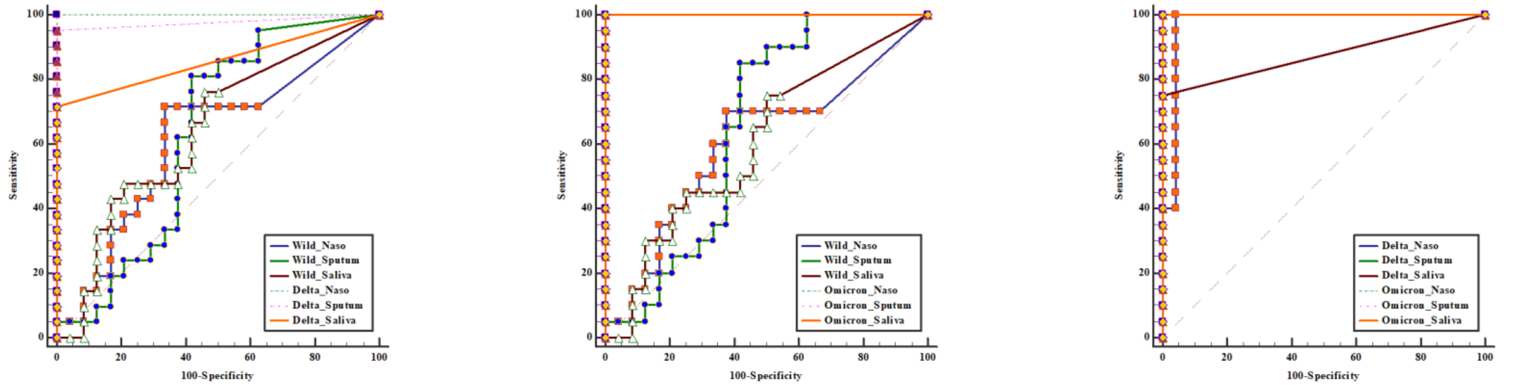

Wild nasopharynx ~ Delta nasopharynx	P < 0.0001
Wild nasopharynx ~ Delta sputum	P = 0.0001
Wild nasopharynx ~ Delta saliva	P = 0.0045
Wild sputum ~ Delta nasopharynx	P < 0.0001
Wild sputum ~ Delta sputum	P < 0.0001
Wild sputum ~ Delta saliva	P = 0.0167
Wild saliva ~ Delta nasopharynx	P < 0.0001
Wild saliva ~ Delta sputum	P = 0.0002

Wild saliva ~ Delta saliva	P = 0.0277
Delta nasopharynx ~ Delta saliva	P = 0.0047
Delta sputum ~ Delta saliva	P = 0.0124
Wild nasopharynx ~ Omicron nasopharynx	P < 0.0001
Wild nasopharynx ~ Omicron sputum	P < 0.0001
Wild nasopharynx ~ Omicron saliva	P < 0.0001
Wild sputum ~ Omicron nasopharynx	P = 0.0001
Wild sputum ~ Omicron sputum	P = 0.0001

Wild sputum ~ Omicron saliva	P = 0.0001
Wild saliva ~ Omicron nasopharynx	P < 0.0001
Wild saliva ~ Omicron sputum	P < 0.0001
Wild saliva ~ Omicron saliva	P < 0.0001
Delta sputum ~ Delta saliva	P = 0.0118
Delta saliva ~ Omicron nasopharynx	P = 0.0118
Delta saliva ~ Omicron sputum	P = 0.0118
Delta saliva ~ Omicron saliva	P = 0.0118

Supplementary Figure legends

Figure S1: Variation in sensitivity for viral strain and sample source.

Comparison of specificity and sensitivity of wild-type, delta, and omicron saliva samples verses nasopharynx and sputum samples.

Figure S2: Pairwise sensitivity comparisons.

Sensitivity and specificity comparison of nasopharynx, sputum, and saliva samples of wild-type vs delta, wild-type vs omicron, and delta vs omicron variants.
